# ATM mediates spermidine-induced mitophagy via PINK1 and Parkin regulation in human fibroblasts

**DOI:** 10.1038/srep24700

**Published:** 2016-04-19

**Authors:** Yongmei Qi, Qian Qiu, Xueyan Gu, Yihong Tian, Yingmei Zhang

**Affiliations:** 1 Gansu Key Laboratory of Biomonitoring and Bioremediation for Environmental Pollution, School of Life Sciences, Lanzhou University, Lanzhou 730000, China; 2 Qibo Medical School, Longdong University, Qingyang 745000, China

## Abstract

The ATM (ataxia telangiectasia mutated) protein has recently been proposed to play critical roles in the response to mitochondrial dysfunction by initiating mitophagy. Here, we have used ATM-proficient GM00637 cells and ATM-deficient GM05849 cells to investigate the mitophagic effect of spermidine and to elucidate the role of ATM in spermdine-induced mitophagy. Our results indicate that spermidine induces mitophagy by eliciting mitochondrial depolarization, which triggers the formation of mitophagosomes and mitolysosomes, thereby promoting the accumulation of PINK1 and translocation of Parkin to damaged mitochondria, finally leading to the decreased mitochondrial mass in GM00637 cells. However, in GM05849 cells or GM00637 cells pretreated with the ATM kinase inhibitor KU55933, the expression of full-length PINK1 and the translocation of Parkin are blocked, and the colocalization of Parkin with either LC3 or PINK1 is disrupted. These results suggest that ATM drives the initiation of the mitophagic cascade. Our study demonstrates that spermidine induces mitophagy through ATM-dependent activation of the PINK1/Parkin pathway. These findings underscore the importance of a mitophagy regulatory network of ATM and PINK1/Parkin and elucidate a novel mechanism by which ATM influences spermidine-induced mitophagy.

Spermidine is a natural polyamine involved in a number of biological processes, such as cell proliferation, the cell cycle and apoptosis^1^,^2^,^3^,^4^. Intracellular spermidine concentration decreases during ageing. However, an exogenous supply can extend lifespan via the activation of autophagy, as demonstrated in several model organisms^5^,^6^. Autophagy selectively and non-selectively eliminates surplus cellular proteins and damaged organelles through the formation of autophagosomes^7^,^8^,^9^,^10^. A well-studied type of selective autophagy is mitophagy, which mediates the removal of damaged mitochondria^11^ and is critical for maintaining proper cellular functions^12^,^13^,^14^. Although it is well established that spermidine can activate certain autophagy-related genes, leading to the activation of autophagy, it is still unknown whether spermidine can induce mitophagy.

The mechanism by which damaged mitochondria are targeted for mitophagy is poorly understood. However, recent studies have shown that mitochondrial priming for selective autophagic recognition is mediated mainly by the PTEN-induced putative kinase 1 (PINK1)/Parkin signaling pathway in most mammalian cells^15^,^16^,^17^,^18^,^19^. As a serine/threonine kinase, PINK1 is selectively stabilized on the outer membranes of defective mitochondria to elicit mitophagy^20^,^21^,^22^. The accumulated PINK1 then recruits the cytosolic E3 ubiquitin ligase Parkin to mitochondria^23^,^24^,^25^,^26^. Subsequently, Parkin ubiquitinates certain proteins on the mitochondrial surface, ultimately leading to the isolation and degradation of defective mitochondria^16^,^27^. However, how this PINK1/Parkin signaling cascade is initiated and whether this pathway is involved in spermidine-induced mitophagy need to be studied further.

The protein kinase ataxia-telangiectasia mutated (ATM), best known for its role as a prime mediator of the DNA damage response^28^,^29^,^30^, is also active in other cell signaling pathways involved in maintaining cellular homeostasis^31^,^32^,^33^,^34^,^35^,^36^,^37^,^38^,^39^. ATM regulates autophagy by activating tuberous sclerosis complex2 (TSC2) or by phosphorylating the transcription regulator hypoxia-inducible factor 1α (HIF1α) to modulate redox homeostasis^40^,^41^,^42^. Other studies found increased numbers of dysfunctional mitochondria in ATM-deficient cells, suggesting that ATM affects mitochondrial homeostasis and quality control^35^,^43^. Nevertheless, it remains rather unclear how ATM executes its specific function in regulating mitophagy.

The present study explores the effects of spermidine on PINK1/Parkin-mediated mitophagy in the SV40-immortalized normal human fibroblast strain GM00637, which contains wild-type ATM, and GM05849 fibroblasts, which are deficient in ATM, to reveal the function of ATM in spermidine-triggered mitophagy. Ultimately, we seek to characterize a novel pathway by which damaged mitochondria are targeted for mitophagy. Our study reveals herein that spermidine induces PINK1/Parkin-mediated mitophagy in ATM-proficient GM00637 cells but not ATM-deficient GM05849 cells. Furthermore, ATM affects the mitophagy process dramatically via regulating the accumulation of PINK1 and the translocation of Parkin. These findings complement the expanding evidence that activated ATM plays versatile functional roles beyond the well-characterized DNA damage response. It triggers a large number of proteins such as PINK1 and Parkin to facilitate mitophagy.

## Results

### Spermidine induced ATM-dependent mitophagy in human fibroblasts

The mitophagic effect of spermidine was determined using measurements of the mitochondrial membrane potential (MMP), analyzing the formation of mitophagosomes and mitolysosomes, and detecting the mitochondrial mass. To determine the proper exposure time and dose of spermidine, cells were exposed to different concentrations of spermidine (10, 50, 100, and 1000 μM) to detect cell viability. In addition, cells were exposed to spermidine for different durations (2, 4, 6, 8, 10 and 12 h) to evaluate the conversion of LC3 I to LC3 II, a well-established marker for autophagy^44^. The results showed that 50 μM spermidine significantly decreased cell viability (supplementary Fig. S1), while increasing the ratio of LC3 II/LC3 I at 2, 4, 6, 8 and 10 h, with the most dramatic effect at 8 h (supplementary Fig. S2). Therefore, 50 μM spermidine was applied to the cells for 8 h, except in the MMP experiments, because it is a relatively early event during mitophagy.

### Spermidine induced mitochondrial depolarization in GM00637 cells

**Figure 1:**
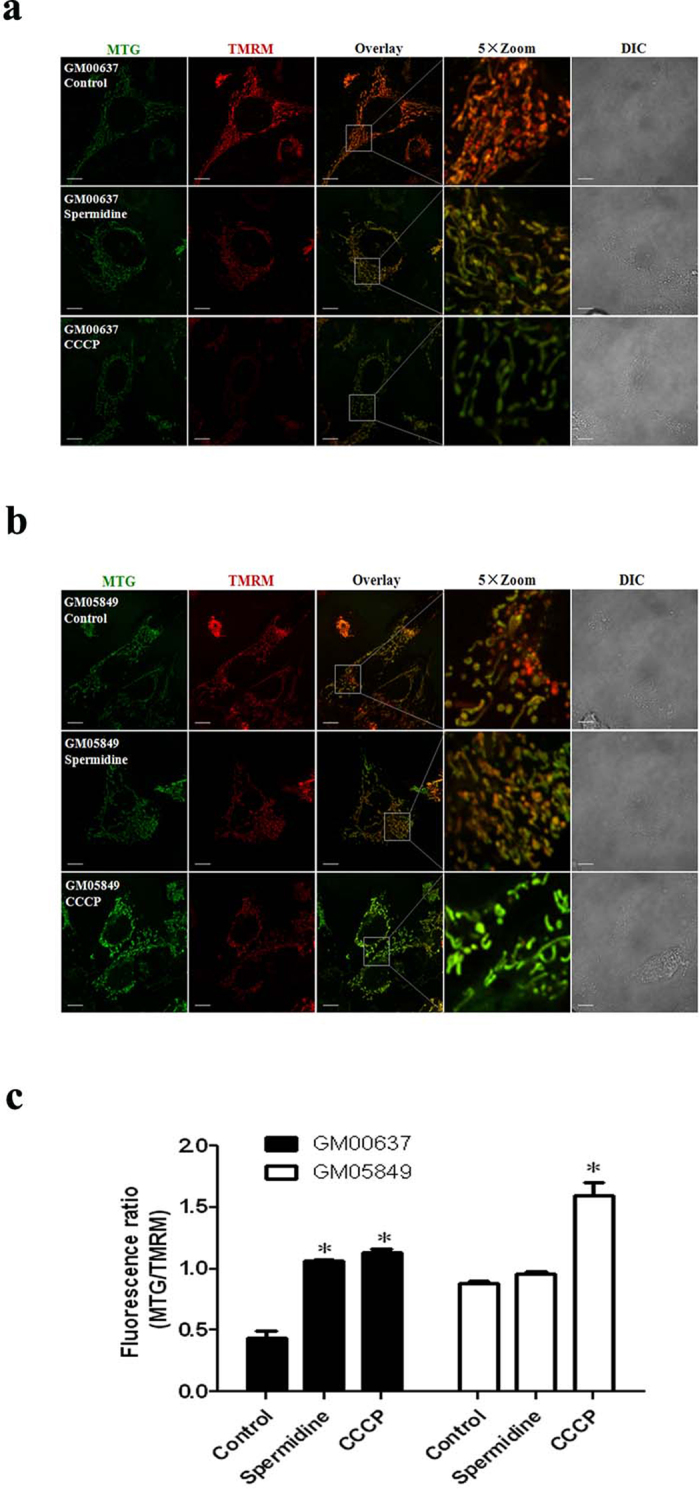
Spermidine induced an ATM-dependent mitochondrial depolarization. The treated GM00637 (**a**) and GM05849 cells (**b**) were stained with TMRM and MTG for 20 min simultaneously and images were taken with a Fluoview FV1000 confocal microscope. The length of the scale bar is 10 μm. Fluorescence intensity was quantified from stacks of images through the entire thickness of cells which were from three independent experiments. MTG/TMRM is indicative of the level of depolarization (**c**). Values are mean ± SD (n = 3), *p < 0.05, compared with control.

To examine the effect of spermidine on MMP, GM00637 and GM05849 cells were treated with 50 μM spermidine for 2 h, followed by incubation with TMRM and MTG (200 nM) simultaneously for 20 min in complete growth medium. Confocal microscopy images revealed an accumulation of MTG and TMRM in the mitochondria, and fluorescence intensity was quantified. In such MTG and TMRM co-loaded cells, depolarizing mitochondria turn from red to green, and the ratio of MTG/TMRM is indicative of the level of depolarization. The control GM00637 cells were in a highly polarized status, as indicated by the stronger fluorescence of TMRM and decreased fluorescence intensity ratio. In contrast, 50 μM spermidine significantly decreased the fluorescence of TMRM (Fig. 1a) and robustly increased fluorescence intensity ratio of MTG/TMRM (Fig. 1c), implying a depolarized status of mitochondria in GM00637 cells. The mitochondria uncoupler CCCP (carbonyl cyanide 3-chlorophenylhydrazone) as a positive control showed a more potent effect. In contrast, GM05849 cells displayed slight depolarization, even in the control group, and spermidine has no obvious effect on MMP as shown by the similar TMRM fluorescence to that of control cells and approximately equal fluorescence intensity ratio of MTG/TMRM (Fig. 1b,c).

### Spermidine induced the formation of mitophagosomes and mitolysosomes and decreased mitochondrial mass in an ATM-dependent manner

**Figure 2:**
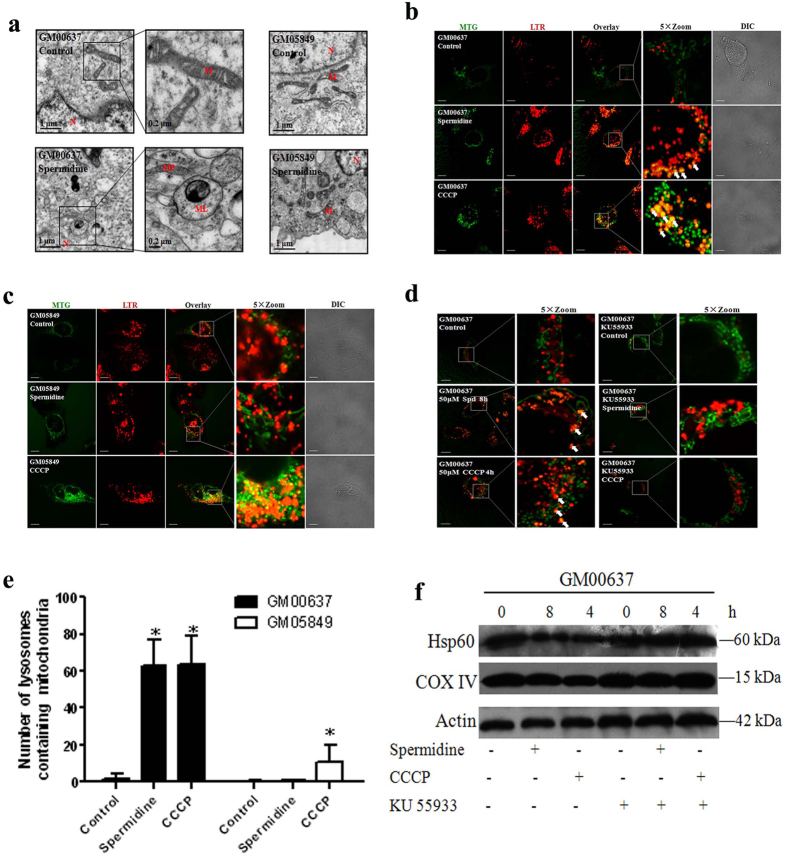
Spermidine induced ATM-dependent formation of mitophagosomes and mitolysosomes and decreased mitochondrial mass. Transmission electron micrographs of GM00637 and GM05849 cells are shown. M: mitochondrion; N: nucleus; MP: mitophagosome; ML:mitolysosome (**a**). Mitochondrial translocation into lysosomes in GM00637 cells (**b**), GM05849 cells (**c**) and KU55933-pretreated GM00637 cells (**d**) are shown. Arrows identify superimposition of green-fluorescing mitochondria with red-fluorescing lysosomes (orange-yellow in color overlay) denoting the formation of mitolysosomes. The scale bar is 10 μm. In (**e**), the number of LTR-labeled lysosomes containing MTG fluorescence is plotted from 20–45 cells/condition. Values are mean ± SD, *p < 0.05 vs. control. Besides, the lysates of GM00637 cells with or without KU55933 pretreatment were immunoblotted using the indicated antibodies (**f**). β-actin was used a loading control.

We next examined the effect of spermidine on mitophagy by direct observation using electron microscopy. GM00637 cells cultured in the presence of 50 μM spermidine for 8 h resulted in a pronounced accumulation of swollen and malformed mitochondria, along with a package of autophagosomes, that formed mitophagosomes. We also observed the monolayer membrane lysosomes engulf the mitophagosomes to become mitolysosomes. Nevertheless, spermidine had no such dramatic effects on the mitochondrial structure in GM05849 cells as indicated by the intact mitochondria (Fig. 2a).

A second method involving the combination of the mitochondria tracker dye MTG and the lysosome tracker dye LTR was used to locate the mitolysosomes. The lysosomes colocalized with mitochondria, denoting the formation of mitolysosomes in spermidine-treated GM00637 cells. Enlarged images showed the lysosome surrounding the mitochondrion (shown by the arrows). CCCP showed more pronounced effects as a positive control (Fig. 2b). However, GM05849 cells (Fig. 2c) or GM00637 cells pretreated with the ATM kinase inhibitor KU55933 (Fig. 2d) abolished mitophagy induction as shown by the significantly decreased formation of mitolysosomes after spermidine treatment. Quantification verified the observation that the total number of LTR-labeled organelles containing MTG-labeled mitochondria was greater in spermidine-treated GM00637 cells than in KU55933-pretreated GM00637 or GM05849 cells (Fig. 2e).

Besides, immunoblotting for mitochondrial matrix protein Hsp60 and inner mitochondrial membrane protein COX IV, which are markers of mitochondrial mass, was used as a quantitative way to monitor the last step of the degradation process of mitophagy. Both Hsp60 and COX IV were dramatically decreased in spermidine-treated GM00637 cells compared with that of control (Fig. 2f), indicating the loss of mitochondria resulting from mitophagy. ATM kinase inhibitor KU55933 compromised this effect as the expressions of these two proteins are not affected when compared with the control group.

### Spermidine induced mitophagy in GM00637 cells through the PINK1/Parkin pathway

**Figure 3:**
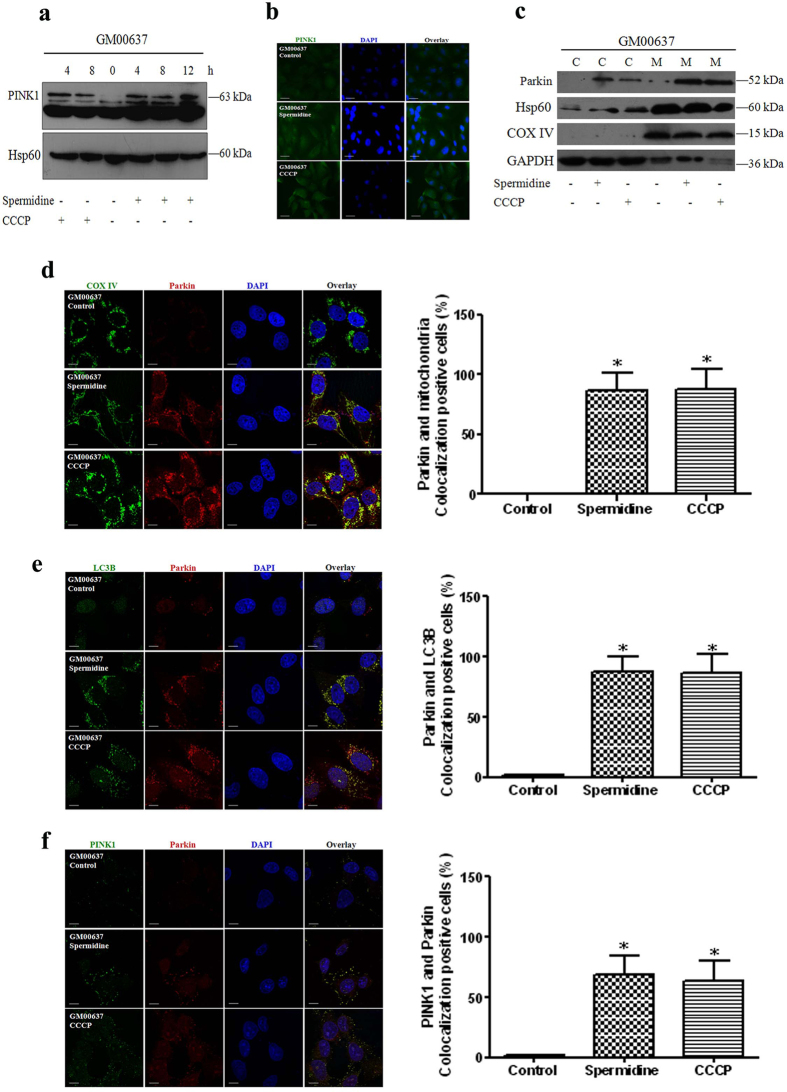
Spermidine affected the accumulation of PINK1 and translocation of Parkin. The lysates of treated GM00637 cells were immunoblotted with anti-PINK1 or anti-Hsp60 (a loading control of mitochondrial proteins) (**a**). Alternatively, the expression of PINK1 in GM00637 cells was detected by immunofluorescence analysis and visualized with an Axio Observer Z1 fluorescence microscope (**b**). The translocation of Parkin was determined by the expression of Parkin after cell fractionation. GAPDH, a loading control of cytoplasmic proteins; Hsp60 and COX IV, loading controls of mitochondrial proteins. C: cytoplasmic fraction; M: mitochondrial fraction **(c).** The colocalizations of Parkin with either mitochondria (labeled by COX IV or Hsp60) (**d**), the autophagosomal marker LC3B (**e**), or PINK1 (**f**) were imaged using a Fluoview FV1000 confocal microscope. The length of the scale bar is 10 μm. Bar graphs show the ratios of Parkin/mitochondria, Parkin/LC3B and Parkin/PINK1 colocalizations from 20–45 cells/condition compiled from three experiments. Values are mean ± SD, *p < 0.05 vs. control.

PINK1 accumulates on the outer membranes of dysfunctional mitochondria when mitophagy is initiated. Under normal conditions, it is rapidly degraded. As shown in Fig. 3a, full-length PINK1 was induced after GM00637 cells were treated with 50 μM spermidine for 4, 8 and 12 h or CCCP for 4 and 8 h. This observation is confirmed by immunofluorescence analysis of PINK1 after treating the cells with 50 μM spermidine for 8 h or CCCP for 4 h (Fig. 3b). Subsequently, PINK1 induced the translocation of Parkin from the cytoplasm to the mitochondria as shown by the increased Parkin expression in mitochondria after cell fractionation (Fig. 3c) and colocalization of Parkin with aggregated mitochondria (labeled by COX IV) (Fig. 3d). Moreover, immunofluorescence analysis revealed that Parkin and endogenous LC3 also colocalized after spermidine (50 μM, 8 h) or CCCP treatments (50 μM, 4 h) (Fig. 3e). Notably, Parkin also colocalized with PINK1 (Fig. 3f). Quantitative analysis of Parkin/mitochondria, Parkin/LC3B and Parkin/PINK1 colocalizations confirmed the above observations.

### ATM regulates PINK1 expression and Parkin translocation during mitophagy

**Figure 4:**
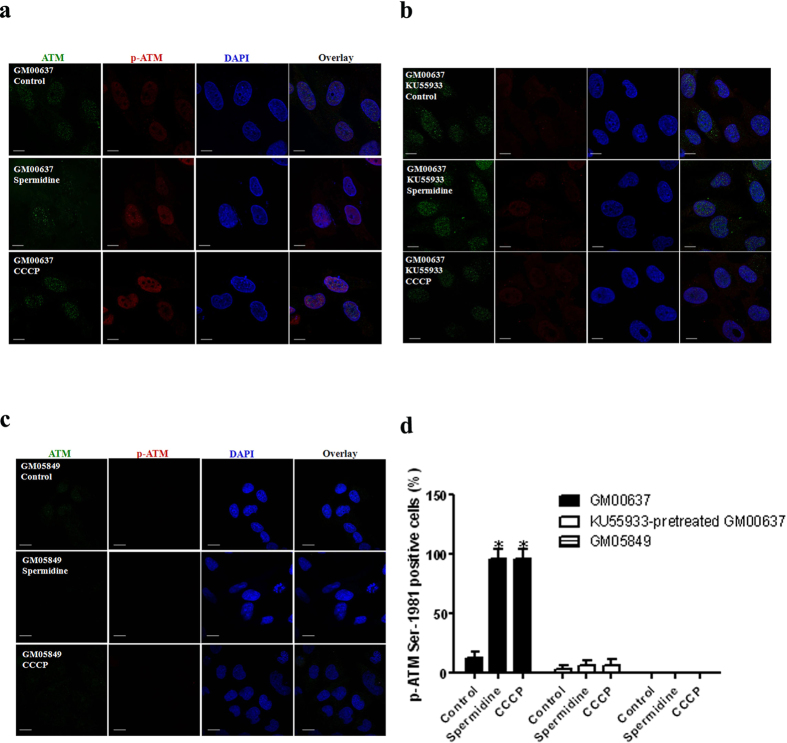
ATM is activated by spermidine in GM00637 cells. GM00637 cells with or without KU55933 pretreatment (**a,b**), and GM05849 cells (**c**) were exposed to 50 μM spermidine or CCCP, followed by immunofluorescence analyses of total and p-ATM on Ser-1981. The scale bar is 10 μm. Ratios of cells expressing p-ATM Ser-1981 to cells expressing total ATM were presented (**d**). 20–45 cells/condition from three experiments were collected. Values are mean ± SD, *p < 0.05 vs. control.

Spermidine-induced mitophagy is dependent on ATM and mediated by the PINK1/Parkin pathway. Therefore, it is critical to determine how this process is regulated. To confirm the role of ATM in spermidine-induced mitophagy, we first examined the phosphorylation of ATM Ser-1981, a general marker of ATM pathway activation. Immunofluorescence analysis showed that p-ATM Ser-1981 levels increased substantially in both the cytoplasm and the nucleus of GM00637 cells (Fig. 4a). The ATM kinase inhibitor KU55933 greatly attenuated the phosphorylation of ATM Ser-1981 (Fig. 4b). Remarkably, ATM is marginally detectable in GM05849 cells (Fig. 4c). The ratios of cells expressing p-ATM Ser-1981 to cells expressing total ATM were recorded (Fig. 4d).

**Figure 5:**
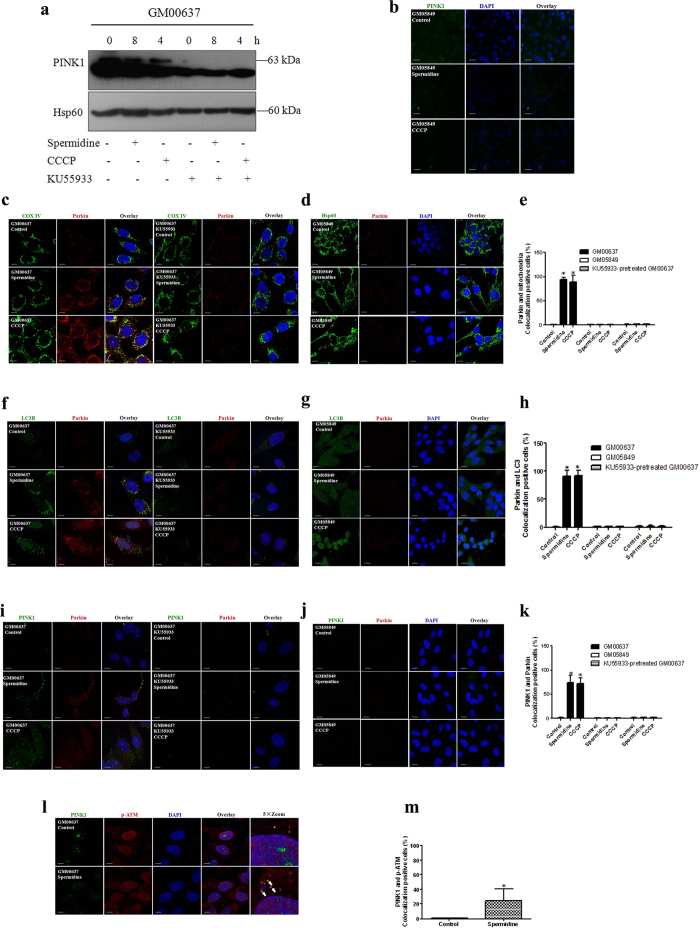
ATM impacted the accumulation of PINK1 and translocation of Parkin. KU55933-pretreated GM00637 cells and GM05849 cells were exposed to spermidine or CCCP. Immunoblotting and immunofluorescence analyses were performed as described in the legend to Fig. 3 (a–k), except for l and m, which show the colocalization of PINK1 and p-ATM Ser 1981.

To further study the involvement of ATM kinase activation in spermidine-induced mitophagy, we determined whether ATM targets the PINK1/Parkin pathway. We show that the expression of PINK1 was inhibited in GM00637 cells pretreated with KU55933 (Fig. 5a) and not detectable in GM05849 cells (Fig. 5b). The translocation of Parkin was blocked in KU55933-pretreated GM00637 cells (Fig. 5c) and GM05849 cells (Fig. 5d). Furthermore, colocalization of Parkin with either LC3 (Fig. 5f,g) or PINK1 (Fig. 5i,j) was also compromised. Notably, spermidine promoted the colocalization of PINK1 and p-ATM (Fig. 5l). The quantitative analyses of Parkin/mitochondria (Fig. 5e), Parkin/LC3B (Fig. 5h), Parkin/PINK1 (Fig. 5k) and PINK1/p-ATM (Fig. 5m) colocalizations fully supported the above observations.

## Discussion

Driven by the discovery that an exogenous supply of spermidine induces autophagy in cultured yeast, nematodes and flies, as well as in a myriad of mammalian cells^6^,^45^, we tested spermidine’s effect on mitophagy in GM00637 and GM05849 human fibroblast cells. Our study demonstrated that spermidine induced mitochondrial depolarization, which is a trigger and a key event in the signaling pathway for mitophagy in ATM-proficient GM00637 cells. Additionally, our transmission electron microscopy analysis revealed that spermidine-treated GM00637 cells formed mitophagosomes and mitolysosomes at the ultrastructural level. Furthermore, fluorescence microscopy analyses revealed that the MTG-labeled mitochondrion colocalized with LTR-labeled lysosomes, and immunobloting analysis demonstrated that the mitochondrial mass (the expression of mitochondrial matrix protein Hsp60 and inner mitochondrial membrane protein COX IV) was decreased in GM00637 cells after spermidine or CCCP treatments. However, ATM deficient GM05849 cells or wild-type GM00637 cells pretreated with the ATM kinase inhibitor KU55933 abrogated spermidine-induced mitophagy. Overall, these results demonstrated that spermidine was not only a potent inducer of general autophagy, but also a strong producer of mitophagy, which is dependent on ATM. This finding is consistent with previous studies proposing that ATM activation is a decisive signal for mitophagy^33^,^46^,^47^.

The mechanisms through which spermidine might exert its mitophagic effect and the exact processes of mitophagy are still not completely known. PINK1 and Parkin, whose mutations are implicated in the onset of Parkinson disease, could be one targeting mechanism because dysfunctional mitochondria specifically accumulate PINK1 and selectively recruit Parkin to promote their autophagy^15^,^17^,^48^. We investigated whether spermidine may affect the PINK1/Parkin pathway. In agreement with earlier reports that the induction of mitophagy following mitochondrial damage is dependent on PINK1and Parkin^25^, we observed that spermidine-treated GM00637 cells underwent a marked effect in this pathway due to the accumulation of PINK1 and the translocation of Parkin. Many studies in the field of mitophagy have colocalized mitochondrial labels and markers of autophagy or other salient proteins such as PINK1 or Parkin^18^,^49^. Here, our results demonstrated that spermidine induced the complete colocalization of Parkin with aggregated mitochondria. Moreover, immunofluorescence analysis clearly revealed that PINK1 and Parkin colocalized after spermidine treatment of GM00637 cells. This result is similar to earlier reports of partial colocalization of wild-type PINK1 with Parkin in mammalian neuronal cells overexpressing both PINK1 and Parkin^23^,^50^. Likewise, Parkin colocalized with endogenous LC3, which is present on isolation membranes and autophagosomes^51^. These results suggest that PINK1 and Parkin function in a signaling pathway to regulate the destruction of defective mitochondria by mitophagy after spermidine treatment of GM00637 cells. Notably, PINK1 expression and Parkin translocation were abrogated in GM05849 cells and KU55933-pretreated GM00637 cells, implying a putative role of ATM in regulating the PINK1/Parkin pathway.

Given that spermidine exerted its mitophagic effect through the PINK1/Parkin pathway, we investigated how the pathway was initiated. A possible component of the targeting process is a signal from the mitochondria that will undergo autophagy. Previous studies and our current study propose that ATM activation is one such signal for mitophagy. Although ATM kinase is best known for its role in orchestrating nuclear DNA damage responses, there is now a growing body of evidence showing that ATM is involved in other important signaling pathways, such as the maintenance of mitochondrial homeostasis^43^,^46^,^47^,^52^. Because mitophagy seems to be impaired in ATM-deficient cells after spermidine treatment, it is tempting to explore whether the ATM kinase regulates the PINK1/Parkin pathway. Our research showed that indeed ATM activated by spermidine promotes the accumulation of PINK1 and translocation of Parkin to mitochondria, thus playing a crucial role in regulating mitophagy. These results support a model in which cells lacking ATM do not undergo PINK1/Parkin-mediated mitophagy. Colocalization of PINK1 with p-ATM strongly suggested that ATM might regulate mitophagy via directly interacting with PINK1. We therefore studied the interaction of PINK1 with total ATM by immunoprecipitation. The results showed that there was no direct interaction between these two proteins (supplementary Fig. S3). However, as a serine/threonine kinase, ATM might regulate mitophagy by phosphorylating PINK1 and Parkin. This intriguing possibility should be explored further.

Because ATM plays such an important role, it is critical to determine how it is activated. Previously, ATM was shown to act as a redox sensor and was activated by oxidative stress in human cells^34^,^53^; our study also found that spermidine induced ATM activation and a reactive oxygen species (ROS) burst in GM00637 cells (supplementary Fig. S4). Therefore, it is reasonable to speculate that ATM is activated by spermidine-generated ROS. A constant increase in ROS was observed after spermidine treatment in GM05849 cells (supplementary Fig. S4). The previous report that the loss of ATM promotes ROS generation supports our ROS observation in these GM05849 cells^34^,^53^.

**Figure 6:**
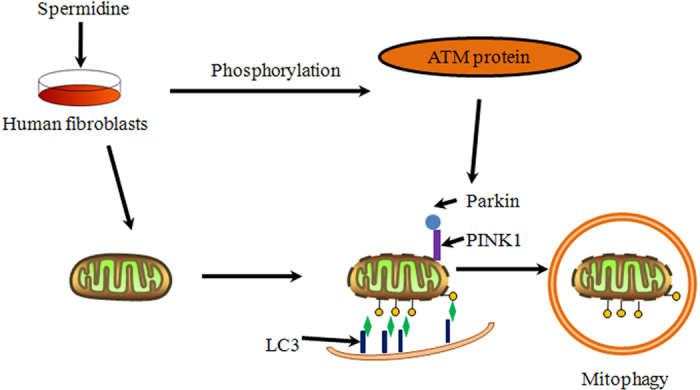
Proposed model of ATM regulating spermidine-induced mitophagy. Spermidine can induce PINK1/Parkin-mediated mitophagy in GM00637 cells. During this process, ATM plays a pivotal role in the accumulation of PINK1 and translocation of Parkin.

In summary, this study demonstrates that the activation of ATM is responsible for PINK1 accumulation and Parkin translocation and subsequent mitophagy after spermidine stimulation (Fig. 6). These findings broaden our understanding of the role of ATM in the mitophagic network.

## Methods

### Chemicals

Spermidine, CCCP and 25% glutaraldehyde were obtained from Sigma-Aldrich Inc. (St. Louis, MO, USA). KU55933 (an ATM kinase inhibitor) was obtained from Tocris (Ellisville, MO, USA). Tetramethylrhodamine methylester (TMRM), MitoTracker^®^ Green FM (MTG) and LysoTracker^®^ Red DND-99 (LTR) were obtained from Life Technologies (Invitrogen, Carlsbad, CA, USA).

### Cell culture

GM00637 and GM05849 cells, a kind gift from Dr. William Bonner’s laboratory (National Institutes of Health, Maryland, USA), were cultured in Dulbecco’s Modified Eagle’s Medium (Gibco, Carlsbad, CA, USA) and Eagle’s Minimum Essential Medium with non-essential amino acids (Gibco, Carlsbad, CA, USA), both of which were supplemented with 10% fetal bovine serum (Royabio, Lanzhou, China), 100 IU/ml of penicillin and streptomycin at 37 °C in a 5% CO_2_ incubator.

### Transmission electron microscopy

GM00637 and GM05849 cells were plated in a 9 cm culture plate and treated with 50 μM spermidine for 8 h. The cells were then collected and fixed with 1 ml of 2.5% glutaraldehyde. The fixative was replaced with fresh liquid every 24 h until the samples were sent to the electron microscope facility of Lanzhou University. The samples were then post-fixed in O_S_O_4_, dehydrated in ethanol and acetone and embedded in resin. Ultrathin sections (60–70 nm) were cut and mounted on pioloform-coated copper grids (Plano). Sections were stained with lead citrate and uranyl acetate and viewed with a transmission electron microscope (JEOL JEM-1230; JEOL Ltd., Japan) operated at 80 kV. Micrographs were taken using a Gatan Erlangshen ES500W camera.

### Fluorescence microscopy and confocal microscopy

GM00637 and GM05849 cells were treated with 50 μM spermidine for 2 h and 50 μM CCCP (positive control) for 30 min. To identify depolarizing mitochondria, the cells were simultaneously stained with 200 nM TMRM and 200 nM MTG for 20 min at 37 °C as described previously^54^. Alternatively, the cells were sequentially incubated with 200 nM MTG and 200 nM LTR for 20 min at 37 °C to stain the mitochondria and acidic organelles such as lysosomes and autophagosomes, as described previously^55^. The images were taken with an Olympus Fluoview FV1000 confocal microscope (Olympus Optical Co., Ltd, Takachiho, Japan). Fluorescence intensity of MTG and TMRM is recorded and the ratio of MTG/TMRM is calculated to indicate the level of depolarization. The number of LTR-labeled lysosomes containing MTG-labeled mitochondria is plotted to denote the mitolysosomes. These data are collected from 20–45 cells which are compiled from three or more independent experiments and the same microscope settings are used between different treatments.

Immunofluorescence analysis was performed as in our previous study^56^,^57^. The treated cells were fixed with 4% paraformaldehyde for 15 min, permeabilized with 90% methanol and 0.3% TritonX-100 for 10 min, followed by incubation with the following antibodies at 4 °C: rabbit anti-ATM (1:500, NB100-104, polyclonal, Novus biological, Littleton, CO, USA); mouse anti-ATM pS1981 (1:500, 200–301–400, monoclonal, Rockland, Gilbertsville, PA, USA); rabbit anti-PINK1 (1:500, ab23707, polyclonal, Abcam, Cambridge, MA, UK); mouse anti-Parkin [PRK8] (1:500, ab77924, monoclonal, Abcam, Cambridge, MA, UK); rabbit anti-LC3B (1:500, L7543, polyclonal, Sigma-Aldrich Inc., St. Louis, MO, USA); rabbit anti-COX IV (1:500, GTX101499, polyclonal, Genetex Inc., Irvine, CA, USA); and rabbit anti-Hsp60 (1:500, GTX110089, polyclonal, Genetex Inc.). Immunostaining was visualized with a fluorescence microscope (Axio Observer Z1, Carl Zeiss, Oberkochen, Germany) or with a confocal microscope (Olympus Fluoview FV1000, Olympus Optical Co., Ltd, Takachiho, Japan). The ratios of cells showing Parkin/mitochondria, Parkin/LC3B and Parkin/PINK1 colocalizations and ratios of cells expressing pATM Ser-1981 to cells expressing total ATM are presented to quantify the expression of indicated proteins. 20-45 cells compiled from three or more independent experiments were collected to obtain the data under the same microscope settings between different treatments.

### Cytoplasmic and mitochondrial protein extraction

Cells were fractionated into cytoplasmic and mitochondrial fractions using Cytoplasmic and Mitochondrial Protein Extraction Kit (Sangon, Shanghai, China). Purity of the fractions was assessed by blotting against GAPDH (cytoplasmic), COX IV and Hsp60 (mitochondrial) proteins. After fractionation, the expression pattern of Parkin was analyzed by western blotting.

### Western blotting

NP-40 Lysis Buffer (Beyotime, Shanghai, China) was used to extract total proteins as in our previous study^19^. After extraction, the proteins were stored at −80 °C for subsequent western blot analysis. The analysis was performed as described previously^57^. Briefly, proteins were separated by sodium dodecylsulfate polyacrylamide gel electrophoresis (SDS-PAGE) and transferred to a polyvinylidene fluoride (PVDF) membrane, and then incubated with primary rabbit antibodies specific to PINK1 (1:1000, ab23707, polyclonal, Abcam, Cambridge, MA, UK), Parkin (1:1000, A0968, polyclonal, ABclonal, College Park, MD, USA), Hsp60 (1:5000, GTX110089, polyclonal, Genetex Inc., Irvine, CA, USA) and GAPDH (1:2000, Zhongshan Golden Bridge Biotechnology, Beijing, China) or mouse antibodies specific to COX IV (1:2000, GTX101499, polyclonal, Genetex Inc., Irvine, CA, USA) and β-actin (1:2000, Zhongshan Golden Bridge Biotechnology, Beijing, China). The samples were incubated with horseradish peroxidase (HRP) -conjugated secondary antibodies (1:20000; ZB-2305 and ZB-2301, Zhongshan Golden Bridge Biotechnology, Beijing, China), and developed with Immobilon Western Chemiluminescent reagents (Millipore, Billerica, MA, USA).

### Statistics

The data were analyzed using GraphPad Prism 5.0 software (GraphPad Software, Inc., La Jolla, CA). Statistical comparisons were made using a one-way analysis of variance (ANOVA) followed by the Least Significant Difference (LSD) post hoc test. Unless indicated otherwise, the level of significance was set at p < 0.05.

## Additional Information

**How to cite this article**: Qi, Y. *et al*. ATM mediates spermidine-induced mitophagy via PINK1 and Parkin regulation in human fibroblasts. *Sci. Rep.* **6**, 24700; doi: 10.1038/srep24700 (2016).

## Supplementary Material

Supplementary Information
