# Supplementary material for: ATM mediates spermidine-induced mitophagy via PINK1 and Parkin regulation in human fibroblasts
Source: Sci Rep. 2016 Apr 19;6:24700. doi: 10.1038/srep24700 (PMC4835770; doi:10.1038/srep24700)
Supplement: Supplementary Information [file srep24700-s1.doc]

ATM mediates spermidine-induced mitophagy via PINK1 and Parkin regulation in human fibroblasts

Yongmei Qi^1#^, Qian Qiu^1, 2#^, Xueyan Gu^1^, Yihong Tian^1^, Yingmei Zhang^1^*

**Supplementary Fig. S1**


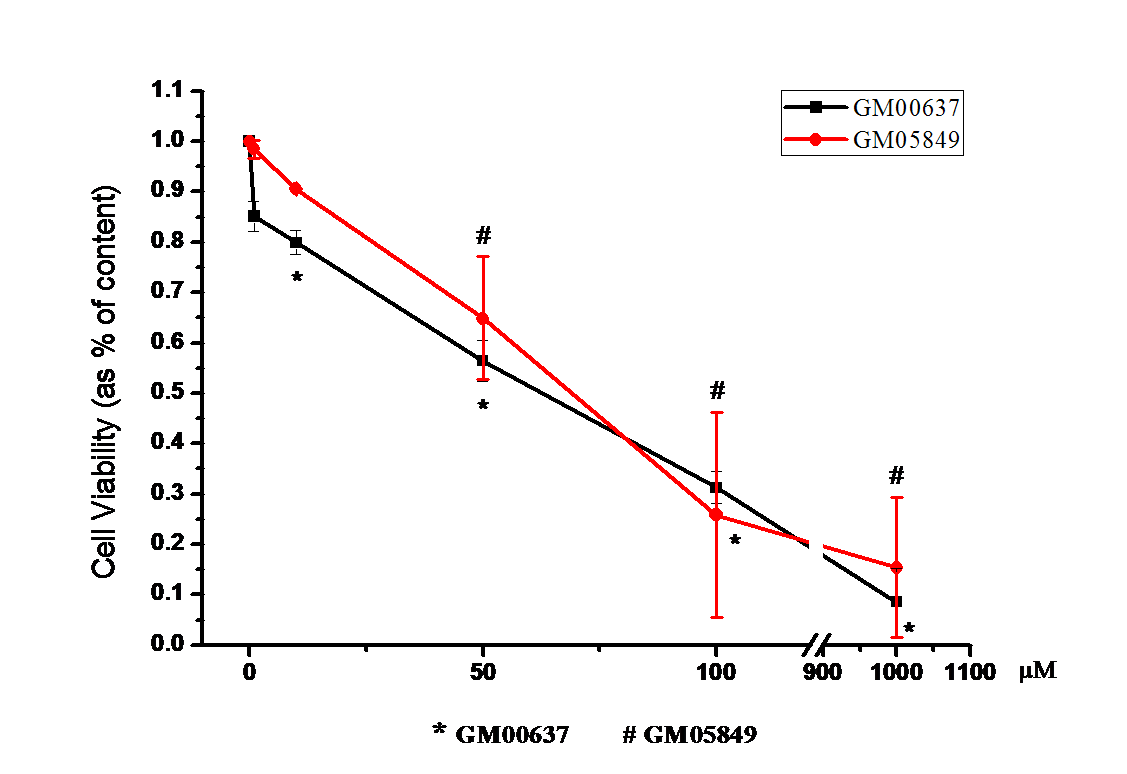


**Supplementary Fig. S2**

a


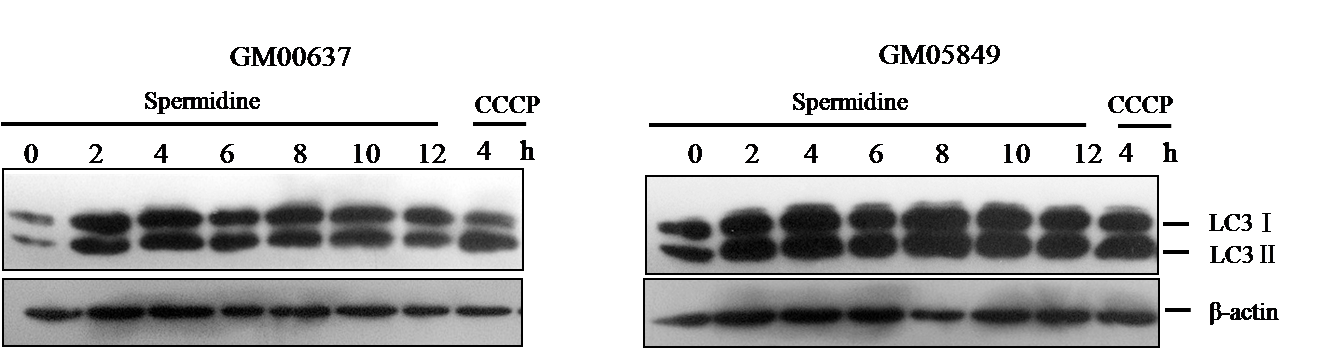


b


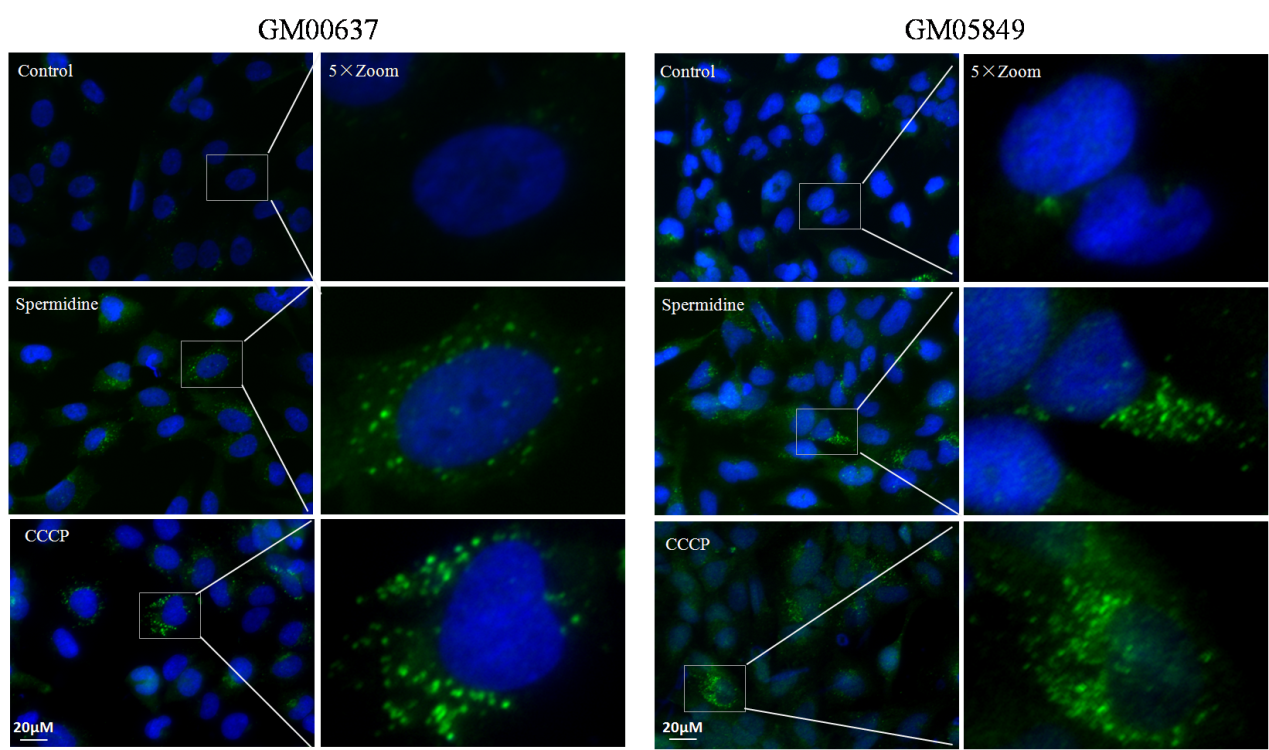


**Supplementary Fig. S3**


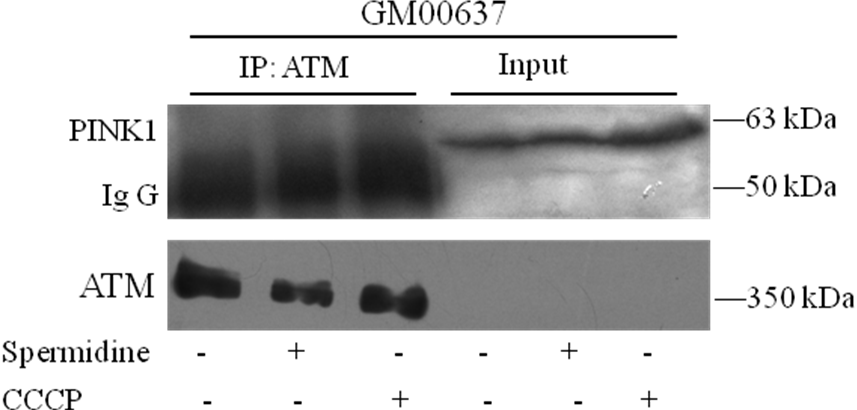


**Supplementary Fig. S4**


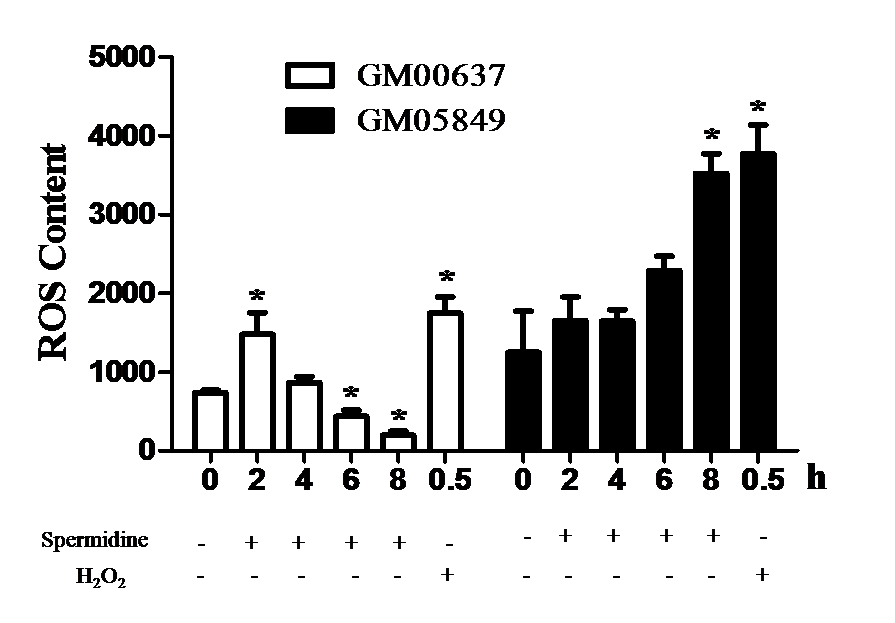

**Supplementary Figure Legends**

**Supplementary Fig. S1** The effect of spermidine on cell viability. GM00637 and GM05849 cells were treated with 0, 10, 50, 100 and 1000 μM spermidine for 24 h. Cell viability was assessed by 3-[4,5-dimethylthiazol-2-yl]-2,5-diphenyltetrazolium

bromide (MTT) assays. Values are mean ± SD (n=3), *, p < 0.05, compared with control of GM00637 cells; #, P < 0.05, compared with control of GM05849 cells.

**Supplementary Fig. S2** Spermidine induced autophagy in human fibroblasts. GM00637 and GM05849 cells were treated with 50 μM spermidine for 2, 4, 6, 8, 10 and 12 h or 50 μM CCCP (positive control) for 4 h. The expression of LC3 protein was analyzed by western blotting (**a**). Alternatively, the cells were treated with 50 μM spermidine for 8 h and followed by immunofluorescence analyses of LC3-II focus (**b**).

**Supplementary Fig. S3** GM00637 cells were treated with 50 μM spermidine for 8 h or 50 μM CCCP (positive control) for 4 h. The lysates were immunoprecipitated (IP) using anti-ATM antibody, followed by immunoblotting using the indicated antibodies. Inputs contained 2% of the amount of lysates used for immunoprecipitation.

**Supplementary Fig. S4** Spermidine induced ROS generation in human fibroblasts. GM00637 and GM05849 cells were exposed to 50 μM spermidine for 0, 2, 4, 6, 8 h, or 200 mM H_2_O_2_ for 0.5 h. After exposure, cells were loaded with 10 μM DCFH-DA for 20 min at 37°C and ROS content was measured by FACS Calibur flow cytometer (Becton Dickinson, San Jose, CA, USA) and analyzed on Cell Quest software. Values are mean ± SD (n=3), *, p < 0.05, compared with control.
